# 

**Published:** 1876-03

**Authors:** 


### CONTENTS:

### ORIGINAL COMMUNICATIONS.

ARTICLE I.—Virginia State Dental Association—Sixth Annual Session........... 481
ARTICLE II.—Address. By W. H. H. Thackston, M. D., D. D. S.................. 498
ARTICLE III—Proceedings of American Academy of Dental Surgery............ 505
ARTICLE IV—On Filling Teeth. By Geo. H. Perine, D. D. S..................... 515

### EDITORIAL, ETC.

Letter from Will J. Kellett, of the N. Y. Press Association..................... 521
To the Dental Profession................................................. 523

### MONTHLY SUMMARY.

Subcutaneous Injection of Water.......................................... 524
Chloride of Lead as a Deodorizer and Disinfectant.......................... 524
Death from Chloroform.................................................... 525
Dislocating the Jaw for Chloroform Narcosis................................ 525
An Excellent Tooth Wash................................................. 525
Bicarbonate of Soda for Toothache........................................ 526
Cancer of Tongue and Jaw................................................ 526
Collodion in Treatment of Carious Teeth.................................. 527
Where to Look for Arsenic in Cases of Poisoning........................... 527
Warts................................................................... 527
Death from Inhalation of Ether........................................... 527
Medical Diploma for Women............................................... 528
Camphor Poisoning....................................................... 528

